# Land Use and Season Interactively Affect Honeybee (*Apis mellifera*) Body Size and Fat Stores

**DOI:** 10.1002/ece3.71889

**Published:** 2025-07-30

**Authors:** Yongqiang Wu, Florian Menzel, Christoph Grüter

**Affiliations:** ^1^ Chinese Academy of Agricultural Sciences Ringgold Standard Institution – Institute of Apicultural Research Beijing China; ^2^ Johannes Gutenberg University Mainz Ringgold Standard Institution – Institute of Organismic and Molecular Evolution Mainz Rheinland‐Pfalz Germany; ^3^ University of Bristol Ringgold Standard Institution – School of Biological Sciences Bristol UK

**Keywords:** body size, fatty acid content, head width, honeybee, land‐using, wing wear

## Abstract

The loss and fragmentation of habitats caused by anthropogenic activities in the last decades has affected foraging habitat quality and, therefore, foraging success (food quantity, quality and foraging range) of many animals, including many pollinators. Honeybees ( *Apis mellifera* ) are important pollinators of many plant species, and habitat change has also affected their ability to collect the resources they need to maintain the colony. Two important biological traits that might be affected by land use are body size and fat stores, which have the potential to affect body condition and therefore success and colony health. However, few studies have investigated these traits in different landscapes and at different times of year. We surveyed 47 sites in three different landscape types (agriculture, urban, and mixed habitats) in south‐western Germany. We measured honeybee body size, wing wear, and analyzed body fat quantity and composition using GC–MS in the spring, summer, and autumn. We found that summer honeybees were smaller in urban and mixed habitats; they showed the greatest wing wear, but they had 18.4%–21.3% larger fat stores compared to agricultural sites. Bees in agricultural habitats experienced a drop in fat stores in summer, whereas body size remained unaffected. In autumn, just before honeybees enter the inactive winter period, bees in urban and mixed areas experienced a drop in fat stores. Wing length decreased from spring to autumn, irrespective of habitat type. Our findings indicate that bees in agricultural settings experience physiological challenges in a central European region in summer, possibly because urban and mixed habitats provide better nutritional conditions during summer. Our findings, thus, confirm that honeybees undergo morphological and physiological changes in response to land use and season, which could impact their physiological condition and winter survival.

## Graphical abstract

Habitat fragmentation and loss due to anthropogenic activities have impacted the foraging success of many animals, including pollinators. In this study, we found that summer honeybees in urban and mixed habitats were smaller and had greater wing wear but had larger fat stores compared to those in agricultural areas, where fat stores dropped during the summer. These findings suggest that land use and season jointly influence bee body size and fat reserves.

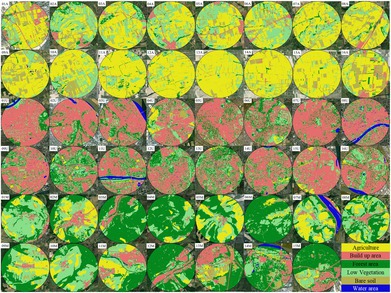

## 1 Introduction

Most flowering plants, including wild and cultivated species, rely on animal pollination (70%–90% of all angiosperm species), and bees, in particular, play important roles as pollinators (Bawa 1990; Fontaine et al. 2005; Ollerton et al. 2011; Potts et al. 2016; Ollerton 2017). In the last decades, however, anthropogenic activity has created landscapes that are increasingly dominated by agricultural monocultures (Aizen et al. 2008; Plourde et al. 2013; Otto et al. 2018) and urban habitat (McDonald et al. 2008; Seto et al. 2012). This conversion of natural habitat into urban or intensively managed agricultural land can lead to food shortages, either in terms of overall quantity or diversity, in some of these landscapes, leading to seasonal foraging challenges, such as a “summer gap” and “green dessert” (e.g., Marcotty 2014; Couvillon et al. 2014b; I'Anson Price et al. 2019; Timberlake et al. 2019). Poor nutrition, in turn, has different negative effects on bees, for example, reduced body size, poor immunity, and lower fat stores (Roulston and Cane 2002; Li et al. 2012; Alaux et al. 2010; Ruedenauer et al. 2020). For example, pollen protein content and diversity were lower in areas of high crop intensification (Donkersley et al. 2014), and honeybee ( *Apis mellifera* ) colonies lost a considerable amount of weight when crops stopped blooming, causing food scarcity and a reduction in individual bee fat stores (Dolezal et al. 2019). The effects of urbanization on food availability appear to be complex (Liang et al. 2023). Some urban areas were found to offer better foraging conditions for honeybees, on the basis of an analysis of foraging distances and flower visitation rates (Theodorou et al. 2020; Samuelson et al. 2021). However, increases in hive numbers in urban areas, due to the increased popularity of urban beekeeping (Alton and Ratnieks 2013; Lorenz and Stark 2015; Stevenson et al. 2020), can potentially lead to a food shortage (Casanelles‐Abella and Moretti 2022). There is evidence that urbanization also affects pathogen loads, with some studies reporting increasing pathogen levels (Youngsteadt et al. 2015; Chau et al. 2023), whereas others show lower pathogen levels in urban habitats (Samuelson et al. 2020).

Another important trait affected by land use is body size: bumblebees (*Bombus*) in urban habitats have been shown to exhibit different body sizes, though these patterns were not consistent across species (Theodorou et al. 2021; Austin et al. 2022). Body size can show considerable intra‐specific variation and is linked to fitness in both solitary and social bees (Goulson et al. 2002; Bosch 2008). Smaller body size can be an indicator of nutritional stress, decreased floral resource availability (Kim 1999), and reduced quality of pollen and nectar in the larval diet (Burkle and Irwin 2009). Stingless bees, for example, adjust worker body size according to hive food stores (Veiga et al. 2013), foraging competition (Segers et al. 2016), and time of year (Quezada‐Euán et al. 2011). Bee size, in turn, can affect foraging ranges, with larger bees foraging at greater distances (Greenleaf et al. 2007; Kendall et al. 2022; Grüter and Hayes 2022).

Fatty acids stored in the bee's fat body—a tissue with an essential role in energy storage, metabolism, and immunity—can also be an indicator of the bee's nutritional condition (Beenakkers et al. 1985; Stanley‐Samuelson et al. 1988). Nutritional stress caused by food shortages can significantly reduce fat stores. For example, Dolezal et al. (2019) found that bees from intensively farmed monocultures experienced reduced fat stores and colony weight when food sources became scarce, both of which can affect survival. Therefore, a better understanding of the links between land use and bee fat stores would help us better understand the links between habitat and bee health.

Nutritional stress has been shown to lead to a range of behavioral changes, such as an early onset of foraging in honeybees (Schulz et al. 1998, 2002) or more intense communication about resources (Rinderer 1982; Wu et al. 2024), thus potentially affecting the foraging load of nutritionally stressed bees. This, in turn, could have an impact on wing damage: bees will acquire and accumulate wing damage because of foraging (Foster and Cartar 2011a) and aging (Mueller and Wolf‐Mueller 1993; Higginson and Barnard 2004), which may further increase foraging effort and reduce lifespan (Schmid‐Hempel and Wolf 1988; Johnson and Cartar 2014; Vance and Roberts 2014) and nectar foraging efficiency (Higginson and Barnard 2004; Foster and Cartar 2011b). Given that land use and season affect the availability and quality of floral resources, it is possible that they also affect the level of wing wear found in honeybee foragers.

**FIGURE 1:**
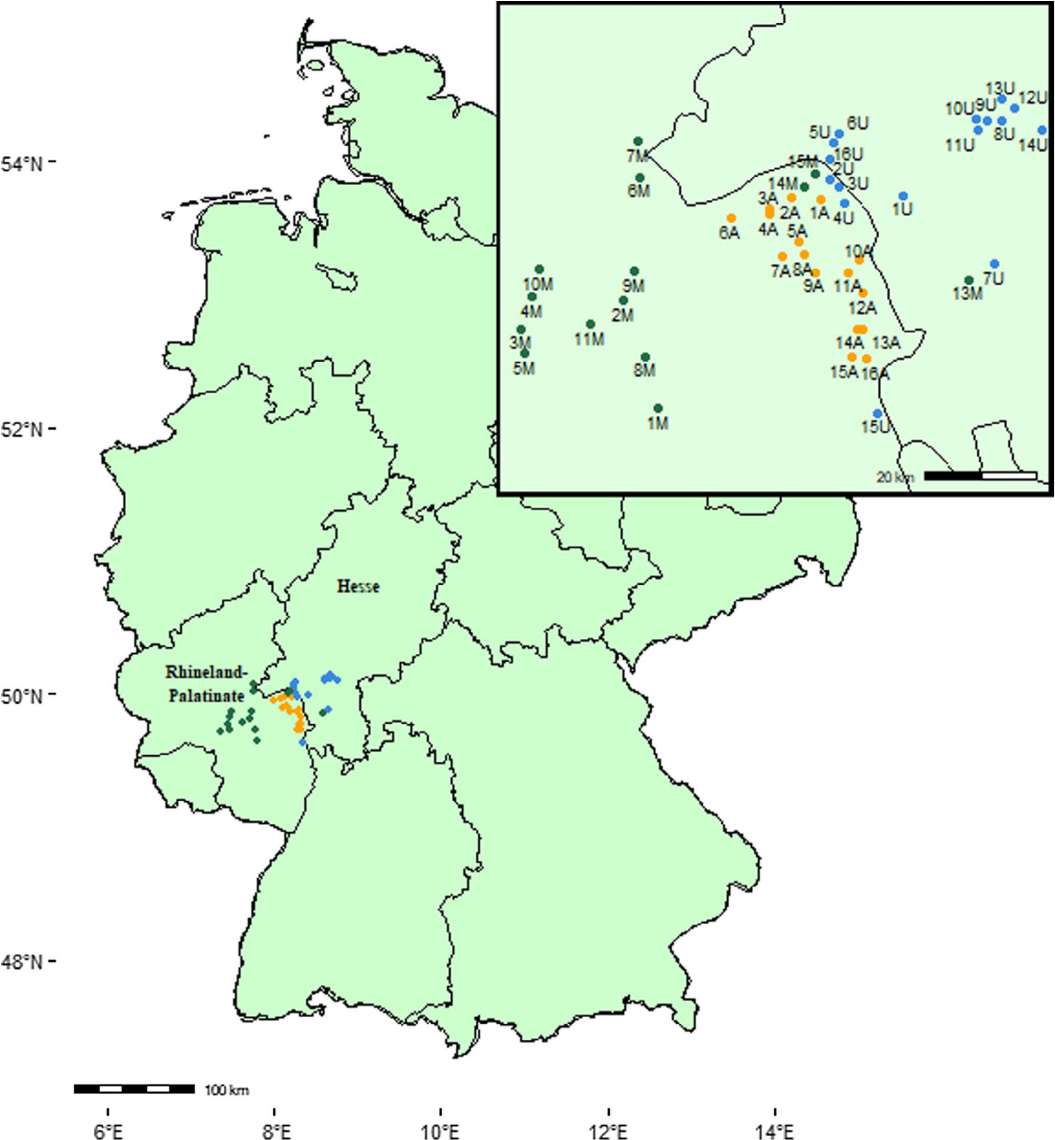
Locations of sampled bees. Dots represent individual sites in the states of Hesse and Rhineland‐Palatinate, south‐western Germany. A (orange) = agricultural landscapes, M (green) = Mixed habitats, U (blue) = urban landscapes.

We aimed to gain a better understanding of how different landscape types and seasons affect the three previously discussed traits with links to nutrition: (1) body size, assessed by measuring head width and wing length, (2) wing wear, a common measure of overall foraging activity (Toth et al. 2009), and (3) bee fatty acid stores, an important physiological trait that has been linked to colony winter survival (Dolezal et al. 2019). We captured free‐flying honeybees visiting flowers in 47 sites in south‐western Germany (states of Hesse and Rhineland‐Palatinate; Figure 1). We focused on three different landscape types: (1) predominantly urban habitats, (2) predominantly agricultural habitats, and (3) mixed habitats. In response to declining insect populations (Steffen et al. 2015; Seibold et al. 2019), some governments have implemented initiatives that provide support for the creation of pollinator‐friendly habitats on agricultural lands (Dicks et al. 2016), such as agri‐environment schemes (AES), which were found to benefit insect biodiversity in Germany (Boetzl et al. 2021) and were particularly attractive for honeybees in the UK (Couvillon et al. 2014a). These programs support insects by supplementing nutritional resources available throughout the season (Scheper et al. 2015; Sidhu and Joshi 2016; Grab et al. 2018). Therefore, our third land use type, “mixed”, included areas that were part of an AES (Kennartenprogramm Rhineland‐Palatinate). We predicted that honeybees captured in these mixed sites are larger and store more fat. We also expected bees to be smaller in summer because of a lack of food sources in many European habitats (Mandelik et al. 2012; Couvillon et al. 2014b; I'Anson Price et al. 2019; Timberlake et al. 2019).

## 2 Materials and Methods

### 2.1 Study Sites and Study Animals

**FIGURE 2:**
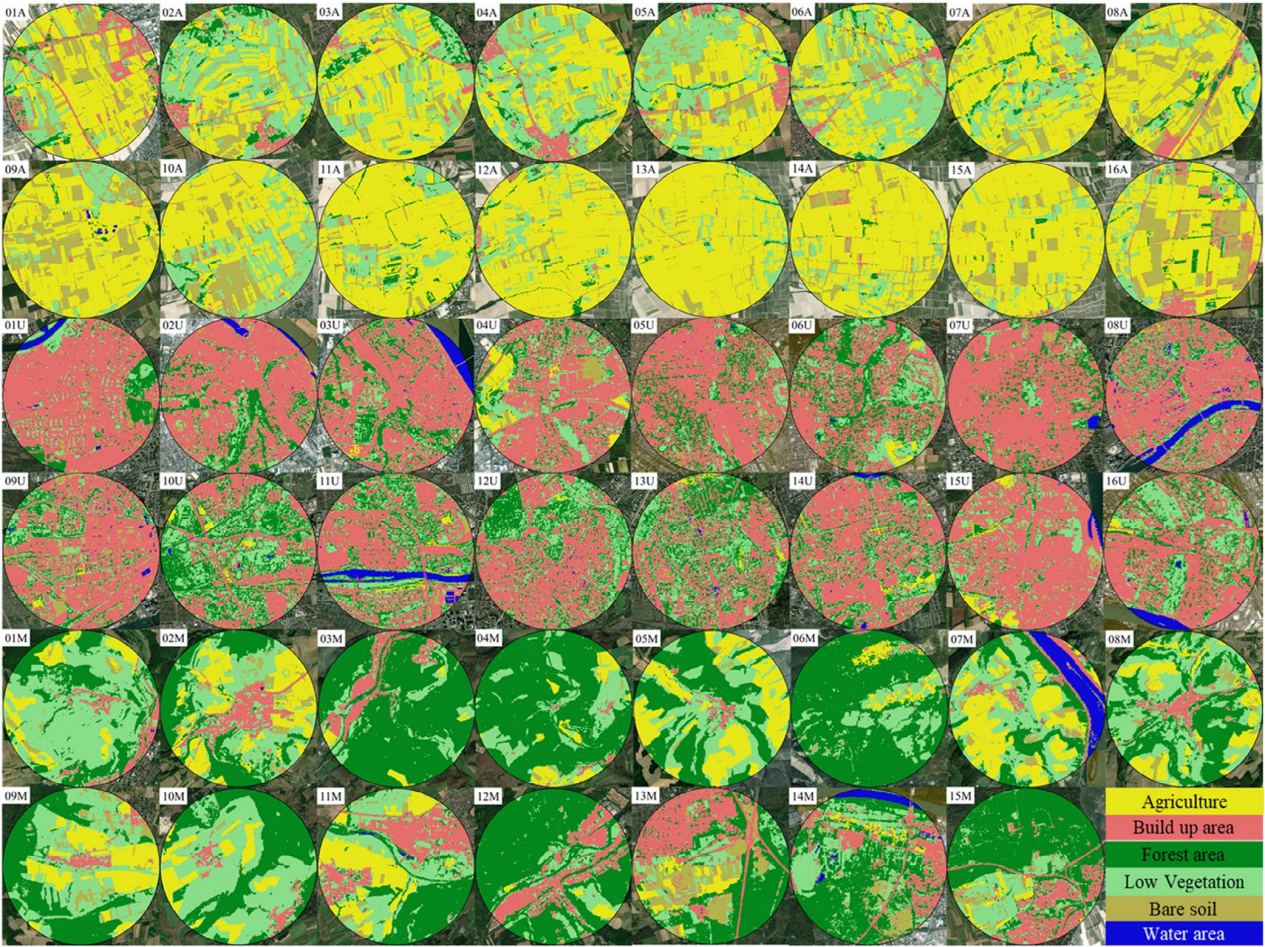
Land use classification of sites (*r* = 1.5 km from the location of bee capture), extracted from the “Landcover Classification Map of Germany 2021 based on Sentinel‐2 Data” (mundialis GmbH and Co. KG 2022): Agriculture (yellow): Cultivated areas, including non‐irrigated and irrigated arable land, crop fields, temporary bare soils (e.g., fallow lands), and areas with vines. Build‐up area (red): Surfaces altered by human construction, replacing natural surfaces with artificial materials (Malinowski et al. 2020). This includes mixed urban and suburban areas like residential, commercial, industrial, transportation, communication, and utilities. Forest area (dark green): Broadleaf tree cover land and coniferous tree cover land. Low vegetation (soft green): Herbaceous vegetation, both natural, low productivity grassland and managed grassland, used for grazing and/or mowing. It also includes low growing vegetation with closed cover and with predominantly shrub and bushy vegetation (limited herbaceous species allowed). Bare soil (moderate yellow): Any natural surface material, including consolidated, mostly impervious surfaces formed by natural materials with a solid surface. This includes surfaces modified by human processes like extraction sites, as well as loose mineral particles from natural sedimentation or human activity, such as mountain slope debris, glacier moraines, river pebble banks, beaches, sand dunes (unvegetated), and quarries. Water area (blue): Water bodies (natural or artificial).

We sampled free‐flying foraging honeybees ( *Apis mellifera* ) at 47 sites located in the states of Hesse and Rhineland‐Palatinate, Germany, from April to October 2021. We distinguished between urban (*N* = 16), agricultural (*N* = 16), and mixed (*N* = 15) sites (Figure 1). Land use data were extracted from the “Landcover classification map of Germany 2021 based on Sentinel‐2 data” (mundialis GmbH and Co. KG (2022); Figure 2 and Table S1). ArcGIS (Version 10.7.1, Esri) quantified land use within a radius of 1.5 km (most foraging happens within this distance from the hive; Steffan‐Dewenter and Kuhn 2003; Couvillon et al. 2014b), with the sampling location as the centre. Urban sites included, for example, the cities of Mainz, Wiesbaden, and Frankfurt and the proportion of build‐up was 55.6% on average (range: 31.3%–72.2%) (U01‐U16; Figure 2; Table S1), whereas agricultural sites (A01‐A16; Figure 2; Table S1) were dominated by agricultural land (on average 57.3%; range: 28.8%–85.3%, mainly growing grapevines (27.5%), wheat (17%), barley (12.8%), and sugar beet (7.8%); Table S8) (Schwieder et al. 2024). Finally, mixed sites (M01‐M15; Figure 2; Table S1) included more diverse types of habitats, including forests (on average 44.5%; range: 19.3%–74.6%), urban habitats (on average 11.1%; range: 1.0%–27.3%), and habitat reserved for the agri‐environment scheme (AES) “Kennartenprogram” of the state Rhineland‐Palatinate. AES have been found to increase insect biodiversity in the neighboring state of Bavaria (Boetzl et al. 2021) (Figure S1). The distance between most sampled locations was at least > 3.0 km (Figure 1), but there were two agricultural sites and seven urban sites with a minimum distance of 2.0 km. Honeybee foraging distances were shorter in urban environments in Samuelson et al. (2021), suggesting that urban hives have smaller foraging ranges.

### 2.2 Bee Collection

We sampled six foraging honeybees at each site in three different meteorological seasons (see below) with a 15 mL Falcon tube. We located bees on flowers, starting our search from the center of the selected sites. Each tube was used to catch one honeybee, and the tubes were immediately put in an insulated portable cooling bag containing reusable ice packs (frozen before use) to stop bees from moving. Cooled bees were kept in a −20°C freezer until further measurements took place. Each site was visited three times corresponding to three different meteorological seasons: once in spring (April and early May), summer (June and July), and autumn (September). Thus, 18 bees were collected at each site. Four of six bees per site and time point were used to measure the fatty acid content, whereas head width and wing measurements were taken from all six bees.

Because of our sampling approach, we do not know if the sampled bees came from wild or managed hives. On the basis of typical abdominal color patterns, we determined that bees belonged to two types, *Apis mellifera carnica* (80%) and Buckfast bee (20%). These ratios were similar in all three land use types: 76%–82% *A. m. carnica*, 18%–24% Buckfast color type. These two types are commonly kept in managed hives by beekeepers in Germany (Ruttner 1988).

### 2.3 Head Width and Wing Length Measurements

Head width (HW, Figure S2) and left forewing length (WL, Figure S3) were used as measures of bees' size, as they reliably correlate with overall body size (Bullock 1999; Grüter et al. 2012; Sauthier et al. 2017). In our study, HW and WL were significantly correlated (Pearson correlation coefficients: *r* = 0.455, *p* < 0.001), and both were considered because land use and season might affect them differently. To further explore morphological variation, we also calculated the head width to wing length (HW/WL) ratio, which provides biological insight into body proportions and may reflect differences in flight morphology and performance (Berwaerts et al. 2002, Spaethe and Weidenmüller 2002). Body parts were placed on laminated graph paper (wings were flattened under a microscope slide) and pictures were taken using an Axiocam 208 microscopy camera mounted on a Stemi 305 (Carl Zeiss, Jena, Germany) stereomicroscope. Subsequently, measurements were performed using ImageJ version 1.54 h (Abràmoff et al. 2004).

### 2.4 Wing Damage

Wing damage can often be found in the form of cuts or missing areas. We classified wing damage using three levels according to Mueller and Wolf‐Mueller (1993): wing margins are (1) hardly damaged (wear < 10%), (2) considerably damaged (10% < wing margins wear < 80%), or (3) extensively damaged 80% < wing margins wear (Figure S4). This classification is based on the proportion of the wing margins affected by damage. We assessed the level of wing wear in honeybee samples in relation to land use type and season.

### 2.5 Fatty Acid Extraction

The most commonly found fatty acids in bee bodies are the *saturated fatty acids* [palmitic acid (C16:0) and stearic acid (C18:0)] and the *unsaturated fatty acids* [palmitoleic acid (C16:1), oleic acid (C18:1), and linoleic acid (C18:2)] (Wu et al. 2024). Palmitic acid, stearic acid, and oleic acid can be biosynthesized by the bees and are most abundant in their bodies. Palmitoleic acid can be converted from palmitic acid in the fat body, but is only present in small amounts. Linoleic acid, on the other hand, has to be acquired from the diet (Stanley‐Samuelson et al. 1988).

Fatty acids were extracted from entire bee abdomens using 1 mL of a chloroform: methanol mixture, 2:1 (v/v) over a period of 24 h (Folch et al. 1957; Wu et al. 2024). The samples were evaporated to dryness under gentle nitrogen flow and then redissolved in 250 μL of a 2:1 dichloromethane: methanol (v/v) mixture. We added 1.6 μg of nonadecanoic acid (dissolved in 10 μL DCM/MeOH) as the internal standard. After vortexing, we moved 5 μL of this solution into a new glass vial and evaporated to dryness under a gentle nitrogen flow. Finally, we added 20 μL trimethylsulfonium hydroxide (TMSH; 0.25 M in MeOH, Sigma‐Aldrich, Munich, Germany) to derivatize the fatty acid methyl esters (FAMEs) and analyzed them with a 7890A gas chromatograph (Agilent) coupled to a 5975C mass‐selective detector (Agilent) (GC/MS). Helium was used as the carrier gas at a flow rate of 1.2 mL per minute. The temperature of the GC oven started at 60°C for 1 min, then increased by 15°C/min to 150°C, followed by an increase to 200°C with a heating rate of 3°C/min, and finally increased by 10°C/min to 320°C, where it was held constant for 10 min. The separated FAMEs were transferred to the MS, and electron ionization mass spectra were recorded at 70 eV from 40 to 650 m/z. Resulting peak areas were integrated manually using the software MSD ChemStation G1701EA E.02.02.1431 (Agilent) and identified on the basis of diagnostic ions, retention time, and the molecular peak. Only fatty acids with abundance > 1% were included in our analyses (Rosumek et al. 2017). This method can detect fatty acids between C10 and C20, but only chain lengths of C16 to C19 were found.

### 2.6 Statistical Analyses

#### 2.6.1 Head Width, Wing Length, and the Ratio Between the Head Width and Wing Length

All data were analyzed in R 4.3.2 (R Core Team 2023). We used general linear mixed‐effects models (LMEs), with sampling sites as a random effect to control for the non‐independence of data from the same sites (bees from one site could be from the same hive) (Zuur et al. 2009). Our fixed effects were *landscape type* (agricultural, urban, and mixed) and *season* (spring, summer, and autumn). We used the “lme4” and “lmerTest” packages for model fitting and estimation of *p*‐values for fixed effects (Bolker et al. 2009). The package “emmeans” was used to estimate *p*‐values for pairwise comparisons (Lenth 2023). We tested if our fixed effects affected the head width and wing length. The head width data caused a singular fit because of low variation in our random effects; therefore, we also used general linear models (LM) without random effects. The *p*‐values for both types of models were very similar, and only LME outputs are shown. The significance of fixed effects and their interaction was tested by comparing models with and without each fixed effect (or interaction) using likelihood ratio tests (LRTs). We used the “rcompanion” package for calculating the means and their confidence intervals (CIs) for fixed effects (Mangiafico 2023). The “DHARMa” (Hartig 2022) package was used to check whether model assumptions were met (Zuur et al. 2009). We used interquartile range (IQR) to check for outliers in the data (Dekking et al. 2005). To evaluate whether spatial autocorrelation may have affected our results, we performed a Moran's I test on bee body traits using the geographic coordinates of our sampling locations. Two distance thresholds were applied to define spatial neighborhoods: 3 km, representing the typical foraging range of honey bees (Steffan‐Dewenter and Kuhn 2003; Couvillon et al. 2014b), and 100 km, corresponding to the maximum distance between sampling sites in our study.

#### 2.6.2 Wing Wear

We analyzed wing damage as an ordinal response variable (1–3) and used Cumulative Link Mixed Models (CLMM). We used the “ordinal” package for model fitting and estimation of *p*‐values for fixed effects (Christensen 2023). Model structure and significance testing followed the procedure described above.

#### 2.6.3 Fatty Acids

We tested for differences in the absolute quantity of fatty acids as well as the proportions of saturated and di‐unsaturated fatty acids (tri‐unsaturated acids were not detected) by normalizing the values using the quantity of the internal standard. The remaining fatty acids, the monounsaturated fatty acids, are equal to 1 (saturated + double unsaturated fatty acids). We used LMEs to compare fatty acid quantities in bees between different seasons and landscapes following the procedure described above. To compare the relative proportions of different types of fatty acids, we used non‐metric multidimensional scaling (NMDS) (command *metaMDS*, package *vegan*) (Oksanen et al. 2024). The permutational multivariate analysis of variance (PERMANOVA) used the *adonis* function (package *vegan*) to assess the significance of “landscapes” and “seasons” as fixed effects while accounting for “sample sites” as a random effect. This was done by randomly rearranging the proportions of different types of fatty acids within the levels of the fixed effects, while keeping the structure of the random effect intact. This approach was used to determine the significance of the observed patterns through permutations.

## 3 Results

### 3.1 Body Size Difference Between Landscapes and Seasons

#### 3.1.1 Head Width Differences in Different Landscapes and Seasons

**FIGURE 3:**
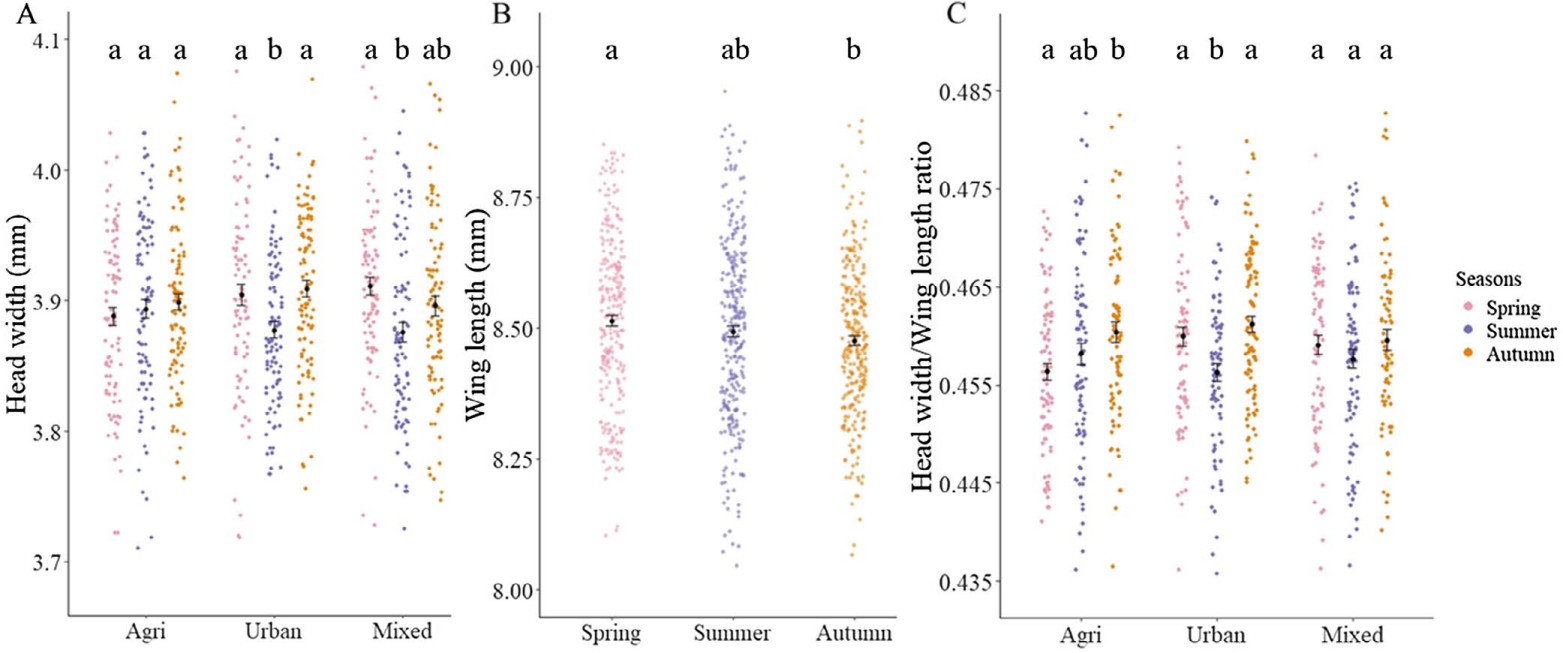
Head width of sampled bees (A). Wing length of bees in the study area (B). The ratio of the Head Width (HW)/Wing Length (WL) (C). Dots represent individual bees in different seasons (pink = spring, blue = summer, and orange = autumn) and landscapes (Agri = Agricultural, Urban = Urban, and Mixed = Mixed habitats). Error bars show the mean value and the standard error. Lowercase letters indicate statistical significance following pair‐wise *t*‐test comparisons (*p* < 0.05).

We measured a total of 840 bees. We found a significant interaction between the fixed effects landscape and season (LME, *LRT* = 11.72, *p* = 0.020); therefore, we separated the data according to landscape to further explore this interaction. In urban sites, bees were smaller in summer than in spring and autumn (Table S2 and Figure 3A). Similarly, bees in mixed habitats were smaller in summer than in spring (HW_Sum_ = 3.88 mm (3.86–3.89) vs. HW_Spr_ = 3.91 mm (3.90 to 3.92), *t* = 3.51, *p* = 0.002; Table S2 and Figure 3A). Autumn bees were intermediate in size (Figure 3A). There was no significant difference between seasons in agricultural areas (LME: df = 2, *F* = 0.63, *p* = 0.53; Table S2 and Figure 3A). There was no significant spatial autocorrelation in head width across sites (Moran's I: 3 km = −0.54179, *p* = 0.706; 100 km = −0.0037, *p* = 0.878).

#### 3.1.2 Wing Length Differences in Different Landscapes and Seasons

We found significant differences in wing length depending on season (LME: df = 2, *F* = 4.08, *p* = 0.017), but not landscape type (LME: df = 2, *F* = 0.15, *p* = 0.86), and there was no significant interaction between landscape and season (*LRTs* = 2.86, *p* = 0.58). Wing lengths were shorter in autumn than in spring (LME: WL_Aut_ = 8.48 mm (8.46–8.49) vs. WL_Spr_ = 8.51 mm (8.49 to 8.53); *t* = −2.85, *p* = 0.012; Table S3 and Figure 3B), with summer bees having intermediate wing lengths (Table S3 and Figure 3B).

#### 3.1.3 Ratio Between Head Width and Wing Length in Different Landscapes and Seasons

We also tested if the ratio between head width and wing length depended on season and landscape type to explore possible morphological changes, with a larger ratio indicating a relatively larger head/shorter wing. We found a significant interaction between landscape and season (*LRT* = 10.86, *p* = 0.03). To explore this further, we analyzed the landscape types separately. We found that in urban habitats, the HW/WL ratio was smaller in summer than in spring and autumn, meaning that summer bees had relatively smaller heads (Table S4 and Figure 3C). In agricultural landscapes, on the other hand, the ratio was larger in the autumn than in the spring (Table S4 and Figure 3C), meaning that autumn bees had relatively smaller wing sizes. There was no seasonal effect in mixed habitats (LME: df = 2, *F* = 1.0, *p* = 0.36).

### 3.2 Wing Wear Depending on Landscape and Season

**FIGURE 4:**
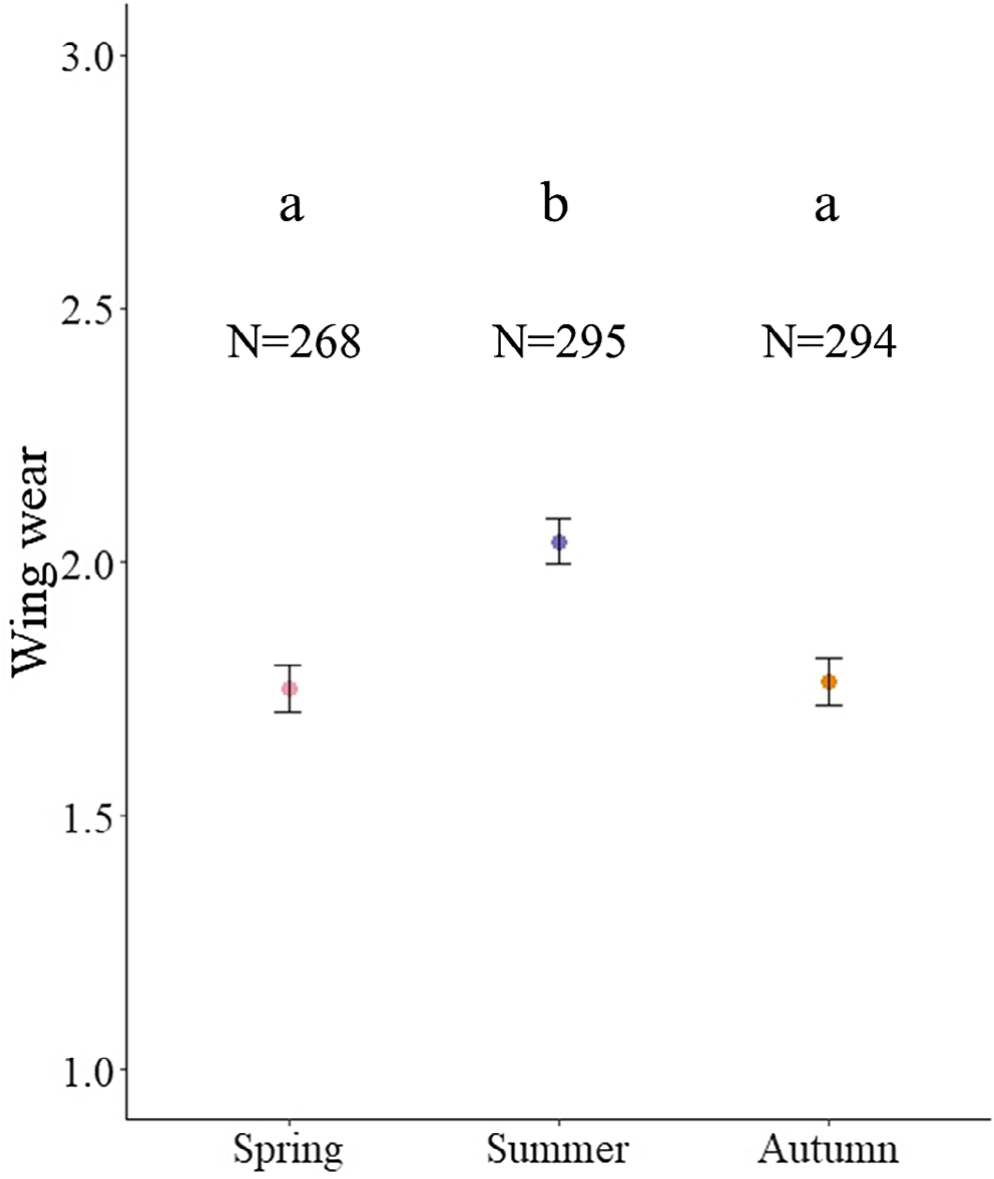
Wing wear in the different seasons. Dots represent the mean, whiskers represent standard errors. a and b lower case letters show a statistical difference (*p* < 0.05) in the tested group.

We found significant differences in wing wear depending on season (*LRTs* = 27.6, *p* < 0.001), but not landscape (*LRTs* = 4.0, *p* = 0.14), and there was no significant interaction between landscape and season (*LRTs* = 4.49, *p* = 0.34). Wing wear was significantly larger in summer compared to spring and autumn (CLMM: *Z* = −3.4, *p* = 0.002; *Z* = −4.9, *p* < 0.001; Figure 4 and Table S5), but there was no significant difference between wing wear in autumn and spring (CLMM: *Z* = 0.034, *p* = 1.0).

### 3.3 Fatty Acid Content in Relation to Landscape and Season

The five main fatty acids identified from the abdomen of individual honeybees were palmitic acid (C16:0), a monounsaturated C16 acid (probably palmitoleic acid, C16:1), stearic acid (C18:0), oleic acid (C18:1), and a diunsaturated C18 acid (probably linoleic acid, C18:2). We analyzed the absolute quantity of fatty acid content and the proportions of different types of fatty acids of honeybees in different landscapes and seasons.

#### 3.3.1 The Absolute Quantity of Fatty Acid Content

**FIGURE 5:**
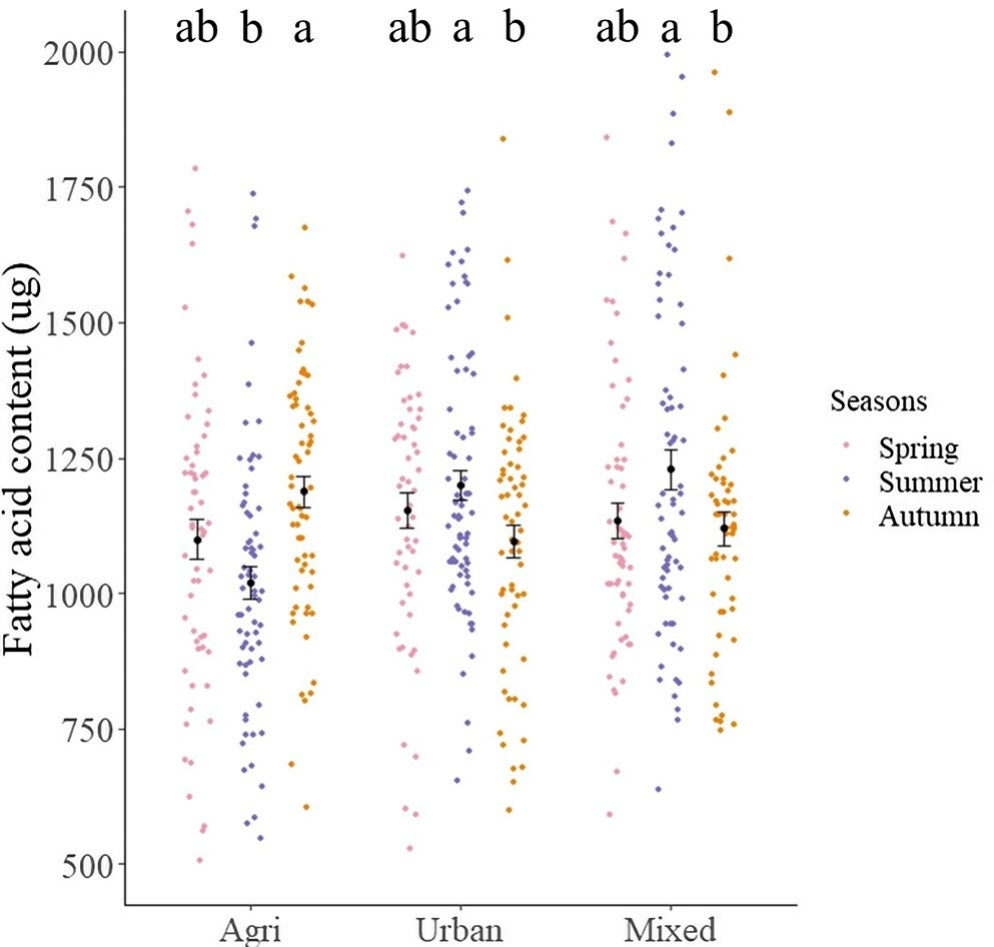
Absolute quantity of fatty acids in the bee abdomen captured in different landscape types (agriculture, urban, and mixed) and seasons (pink = spring, blue = summer, and orange = autumn). Dot and whisker represent the mean and the standard error, respectively. Lowercase letters indicate statistical significance following pair‐wise *t*‐test comparisons (*p* < 0.05).

We found a significant interaction between landscape type and season (LME: *LRT* = 29.41, *p* < 0.001). Therefore, we analyzed the different landscapes separately. In agricultural landscapes, we found a lower quantity of fatty acids (16.3%) in summer than in the autumn (*t* = −3.82, *p* < 0.001; Table S6 and Figure 5), with spring bees having intermediate levels of fatty acids. In contrast, we found that fatty acid content was higher in summer than in autumn in urban and mixed landscapes (9.1% and 9.7%, respectively) (Table S6 and Figure 5). When separating the different seasons, we found that fatty acid content was lower in agricultural sites than in urban (18.4%) and mixed (21.3%) sites in summer, but there was no significant difference in spring and autumn (Table S6 and Figure 5).

#### 3.3.2 The Proportion of Different Types of Fatty Acids in Different Landscapes and Seasons

We found significant differences in the proportion of di‐unsaturated C18 acid (probably linoleic acid, C18:2) between seasons (LME: df = 2, *χ* ^2^ = 485.36, *p* < 0.001), but not landscapes (LME: df = 2, *χ* ^2^ = 4.24, *p* = 0.12). There was no interaction between the landscape and season (*LRTs* = 6.48, *p* = 0.17). Bees had a higher proportion of doubly unsaturated fatty acids in spring than in summer and autumn (Figure S5 and Table S7). Furthermore, we found significant differences in the proportion of monounsaturated fatty acids between seasons (LME: df = 2, *χ* ^2^ = 219.11, *p* < 0.001), but not between the landscapes (LME: df = 2, *χ* ^2^ = 0.60, *p* = 0.74). There was no interaction between the landscapes and seasons (*LRTs* = 4.47, *p* = 0.35). The proportion of monounsaturated fatty acids was lower in spring than in summer and autumn (Table S7). However, we found no significant differences in the saturated fatty acids depending on landscape (LME: df = 2, *χ* ^2^ = 1.31, *p* = 0.52) and season (LME: df = 2, *χ* ^2^ = 1.83, *p* = 0.40), and there was no significant interaction between landscape and season (*LRTs* = 5.72, *p* = 0.22).

### 3.4 Relationship Between Head Width and Fatty Acid Content in Honeybee

We also tested whether the total fatty acid content of a bee is related to body size. However, we found no significant relationship between head width and absolute fatty acid content (LME: df = 1, *χ* ^2^ = 0.2465, *p* = 0.62).

## 4 Discussion

We found that land use type and season affected bee size and fat stores, often interactively. As predicted, bees had smaller heads in summer compared to spring and autumn, but this was only found in urban and mixed habitats, whereas bee size remained constant in agricultural sites (Figure 3A). In contrast, we found that wing lengths decreased from spring to autumn (Figure 3B). Summer is known to be a challenging period for colonies in temperate European habitats, both agricultural and urban, because of a scarcity of flowers (Nürnberger et al. 2017; Dolezal et al. 2019; I'Anson Price et al. 2019; Czekońska et al. 2023) before the bloom of ivy ( *Hedera helix* ) in autumn improves the foraging conditions for bees (Garbuzov and Ratnieks 2014; Knoll et al. 2024). The relative scarcity of food sources during summer may force bees to forage further away from their hives (Couvillon et al. 2014b). This might explain why wing wear was highest in summer. Wing wear directly impacts the flight ability and reflects cumulative foraging effort (Mueller and Wolf‐Mueller 1993). There is evidence that increased wing wear reduces the lifespan and foraging efficiency of honeybees (Foster and Cartar 2011b; Vance and Roberts 2014). The challenging summer conditions are consistent with our finding that honeybees were smaller in summer, but it is somewhat unexpected that this effect was only found in urban and mixed landscapes, not in agricultural landscapes (Figure 3A). One explanation could be that some agricultural areas experience a short‐term feast in late spring and early summer (Dolezal et al. 2019), benefitting bee size later in summer. Alternatively, producing smaller bees in summer might allow colonies that do well to increase brood production rate and boost colony population (Ramalho et al. 1998). This could reflect different investment strategies in response to food resource availability in spring and early summer (Kim and Thorp 2001) and deserves further study. Furthermore, high temperatures in summer might affect larval growth rate and, thus, the body size of bees: Kelemen and Rehan (2021) found that individuals of *Ceratina calcarata* were smaller when reared in warmer temperatures (see also Sibly and Atkinson 1994).

The relatively large size of spring bees should be interpreted with caution because the long lifespan of winter bees (Fukuda and Sekiguchi 1966; Smedal et al. 2009) means it is possible that bees collected in spring were actually winter bees that developed in autumn of the previous year. As mentioned before, foraging conditions are often good in autumn because of the availability of ivy flowers, leading to an improved larval diet for the production of winter bees (Garbuzov and Ratnieks 2014; Knoll et al. 2024). In contrast, Sauthier et al. (2017) found a general tendency for bees to become larger during the foraging season, but their study included only two sites (compared to our 47), and their results could be driven by local foraging conditions. Our results also suggest that trait similarity was not structured by geographic proximity, and our observed patterns are unlikely to be driven by an underlying environmental gradient or spatial cline. Consistent with this, we also found that the two land use types that are most geographically distant, mixed and urban, exhibited similar trait values, further supporting the absence of spatial structuring.

The reduction of wing length over time was unexpected (Figure 3B) and future research could explore if this impacts the flight performance of bees. This could be the result of an accumulation of pathogens or an increase in temperature fluctuations (Es'kov and Es'kova 2013; Janczyk and Tofilski 2021; Tafi et al. 2024). Our results also contrast with the findings of Es'kov and Es'kova (2013), who found that honeybees in a Russian habitat increased in wing size by the end of summer (Table S3 and Figure 3B). To further explore these morphological changes, we studied the effects of landscape type and seasons on the ratio *head width/wing length* (HW/WL). We found that in urban areas, the HW/WL ratio was smaller in summer than in spring and autumn (Table S4 and Figure 3C), indicating that summer bees had relatively smaller head size in urban environments. In agricultural landscapes, on the other hand, autumn bees had relatively smaller wings (Table S4 and Figure 3C).

Our data on fat stores again revealed that bees in agricultural sites show different patterns than bees in urban and mixed habitats. However, the direction of the effect was the opposite of what we expected. Although being smaller, summer bees stored more fat in urban and mixed habitats (18.4% and 21.3%, respectively) than in agricultural habitats (Figure 5). Urban and mixed habitats are likely to offer a greater diversity of food in summer (Danner et al. 2017; Baldock et al. 2019; Tew et al. 2021), which could allow bees to store more fat in these habitats. In autumn, however, bees carried 9% less fat in urban and mixed areas. Conversely, in agricultural habitats, bees increased their fat content by 16.3% from summer to autumn, achieving fat levels similar to those of bees in urban and mixed habitats (Figure 5). These results highlight the lack of a positive correlation between bee size and fat stores. Several reasons could explain why bees in urban and mixed habitats store more fat than bees in agricultural areas during summer. Firstly, urban and mixed areas may provide pollen types with higher protein content. Donkersley et al. (2014) found that pollen protein content was lower in arable and horticultural farmland and correlated positively with the presence of natural grassland, broadleaf woodlands, and built‐up areas. This, however, might change in autumn when urban and forested land covers offered the least valuable sources for pollinators in a study by Richardson et al. (2023). In agricultural areas, bees might experience an increase in pollen foraging diversity after the summer gap and before winter, helping them to build up more fat stores (Knoll et al. 2024). Another explanation for the increase in fat stores in agricultural areas could be that beekeepers in these areas feed their hives more in autumn (when feeding typically happens) compared to beekeepers in urban or mixed areas. Even though a previous study found that honey stores did not affect bee fat stores in the medium term (Wu et al. 2024), we cannot rule out the effects of differences in bee husbandry. For example, Dolezal et al. (2016) found that *Varroa* mite infestation also affected lipid levels. Bees in landscapes of low cultivation had higher lipid levels in autumn compared to those in areas with high cultivation, but this pattern was observed only in colonies free of *Varroa* mites. This finding suggests that differences in mite prevalence, for example, because of differences in mite treatment practices among beekeepers, may have influenced the lipid levels of our bees.

The most common fatty acids in bees, including both saturated and unsaturated fatty acids, are stored in fat, and only about 5% are components of cell membranes (Stanley‐Samuelson et al. 1988; Ruess and Chamberlain 2010). Palmitoleic acid (C16:1), oleic acid (C18:1), and linoleic acid (C18:2) are additionally related to antimicrobial defense and cognitive functions (Ramanathan et al. 2018; Arien et al. 2018; Kim et al. 2020; Domínguez et al. 2024). We found that the doubly unsaturated fatty acids (most likely linoleic acid, which can only be acquired through the diet, Rosumek et al. 2017; Arien et al. 2020) were 47%–49% higher in spring than in summer and autumn, whereas the monounsaturated fatty acids (probably palmitoleic acid, C16:1, and oleic acid C18:1) were 8% lower in spring than in summer and autumn (Figure S5 and Table S7). Our findings align with the general observation that increasing dietary polyunsaturates are associated with higher proportions of polyunsaturated fatty acids and lower proportions of monounsaturated fatty acids in tissues (Stanley‐Samuelson et al. 1988). This suggests that honeybees collect and consume more pollen that includes significant amounts of linoleic acid, such as dandelion (14% linoleic acid) in spring (Standifer 1966).

## 5 Conclusions

We found that landscape and season interactively affect honeybee body size, wing wear, and fat stores. We found that in summer, bees in these areas experience greater nutritional and physiological challenges compared to bees in urban and mixed habitats. Despite their slightly smaller body size, bees in urban and mixed habitats may experience more favorable conditions in summer, as evidenced by increased fat stores. This supports the view that urban and mixed habitats can be a refuge during the particularly challenging summer months. More research is needed to understand the behavioral and health implications of our findings. Our findings suggest that to improve overwintering success and prevent colony losses, management decisions should aim to increase the nutritional diversity and availability of food for bees: (1) in agricultural habitats during early summer and (2) in urban and mixed habitats at the start of autumn.

## Author Contributions

**Yongqiang Wu:** conceptualization (equal), data curation (lead), formal analysis (lead), funding acquisition (lead), investigation (lead), methodology (lead), project administration (equal), software (lead), validation (equal), visualization (lead), writing – original draft (lead). **Florian Menzel:** conceptualization (equal), data curation (supporting), formal analysis (supporting), methodology (supporting), software (lead), supervision (equal), writing – review and editing (equal). **Christoph Grüter:** conceptualization (lead), data curation (lead), formal analysis (equal), investigation (equal), methodology (lead), software (supporting), supervision (lead), visualization (equal), writing – original draft (equal), writing – review and editing (equal).

## Conflicts of Interest

The authors declare no conflicts of interest.

## Supporting information


**Figure S1.** Blue area represents the agri‐environmental scheme (AES). Pink dots show our study sites. Black dots show the cities around the study sites.
**Figure S2.** The head width (mm) of the bees (3‐17A‐12).
**Figure S3.** The wing length (mm) of the bees (3‐03A‐01).
**Figure S4a.** A level: wing margins wear < 10% (3‐03A‐01).
**Figure S4b.** B level: 10% < wing margins wear < 80% (1‐12 M‐06).
**Figure S4c.** C level: 80% < wing margins wear (1‐05 M‐10).
**Figure S5.** Proportion of polyunsaturated fatty acids in the captured bee abdomen. Dot and whisker represent the mean and the standard error, respectively. Lowercase letters indicate statistical significance following pair‐wise t‐test comparisons (*p* < 0.05).

**Table S2** Head width (mm) differences in different seasons and landscapes.
**Table S3.** Wing length (mm) differences in different seasons.
**Table S4.** Ratio between wing length and head width in different landscapes and seasons.
**Table S5.** Wing wear differences in different seasons.
**Table S6.** Absolute fatty acid content (AbsFA) in different landscapes and seasons.
**Table S7.** Proportion of each fatty acid content in different seasons.

**Table S1.** Proportions of different land‐cover types around sampled Sites (2021).

**Table S8.** Agricultural land use categories (2021).

## Data Availability

The data set used for this study is available in the Supporting Information.
