# Supplementary material for: Land Use and Season Interactively Affect Honeybee (Apis mellifera) Body Size and Fat Stores
Source: Ecol Evol. 2025 Jul 30;15(8):e71889. doi: 10.1002/ece3.71889 (PMC12310833; doi:10.1002/ece3.71889)
Supplement: Supplementary file 1 — Figure S1. Blue area represents the agri‐environmental scheme (AES). Pink dots show our study sites. Black dots show the cities around the study sites. Figure S2. The head width (mm) of the bees (3‐17A‐12). Figure S3. The wing length (mm) of the bees (3‐03A‐01). Figure S4a. A level: wing margins wear < 10% (3‐03A‐01). Figure S4b. B level: 10% < wing margins wear < 80% (1‐12 M‐06). Figure S4c. C level: 80% < wing margins wear (1‐05 M‐10). Figure S5. Proportion of polyunsaturated fatty acids in the captured bee abdomen. Dot and whisker represent the mean and the standard error, respectively. Lowercase letters indicate statistical significance following pair‐wise t‐test comparisons (p < 0.05). [file ECE3-15-e71889-s003.docx]

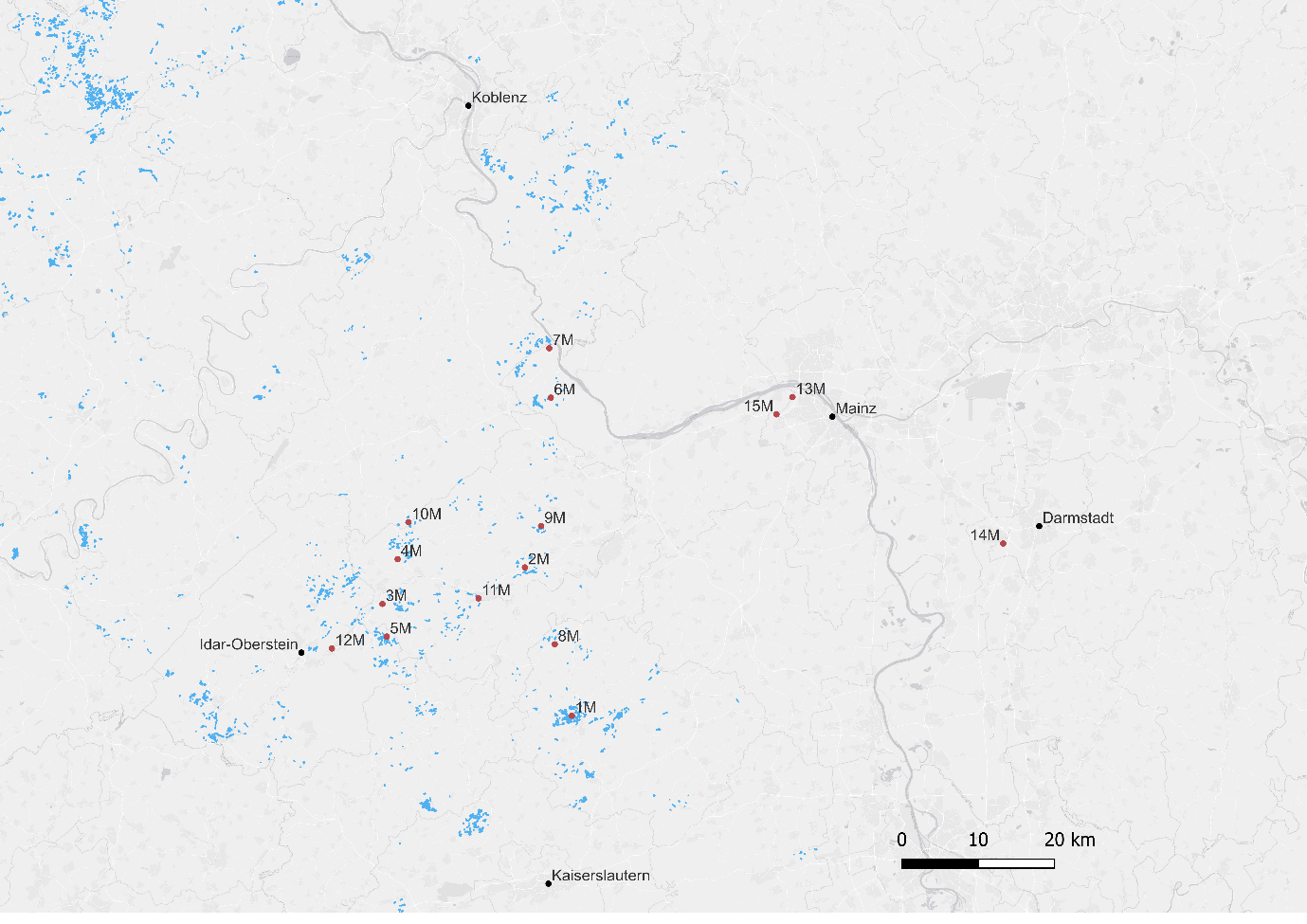


**Fig S1**. Blue area represent the agri-environmental scheme (AES). Pink dots show our study sites. Black dots show the cities around the study sites.


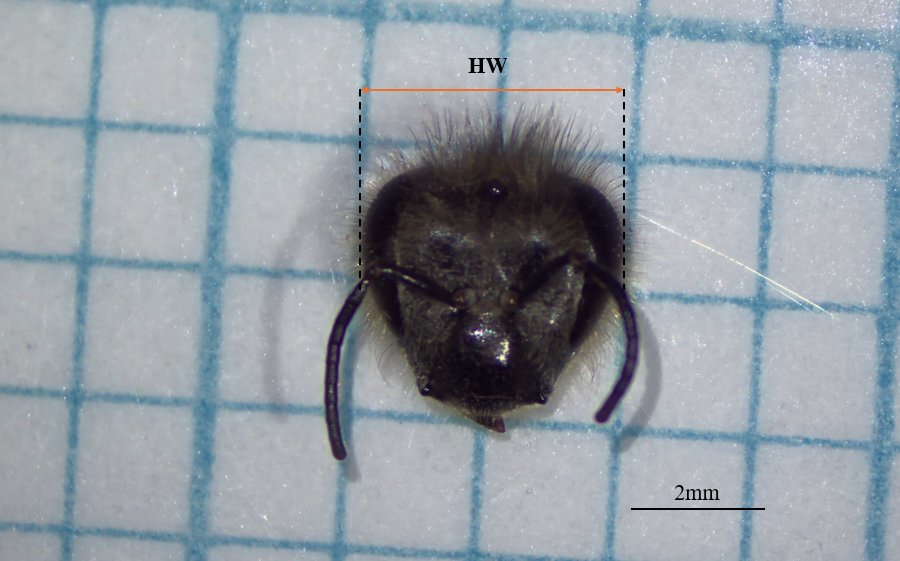


**Fig S2**. The head width (mm) of the bees (3-17A-12).


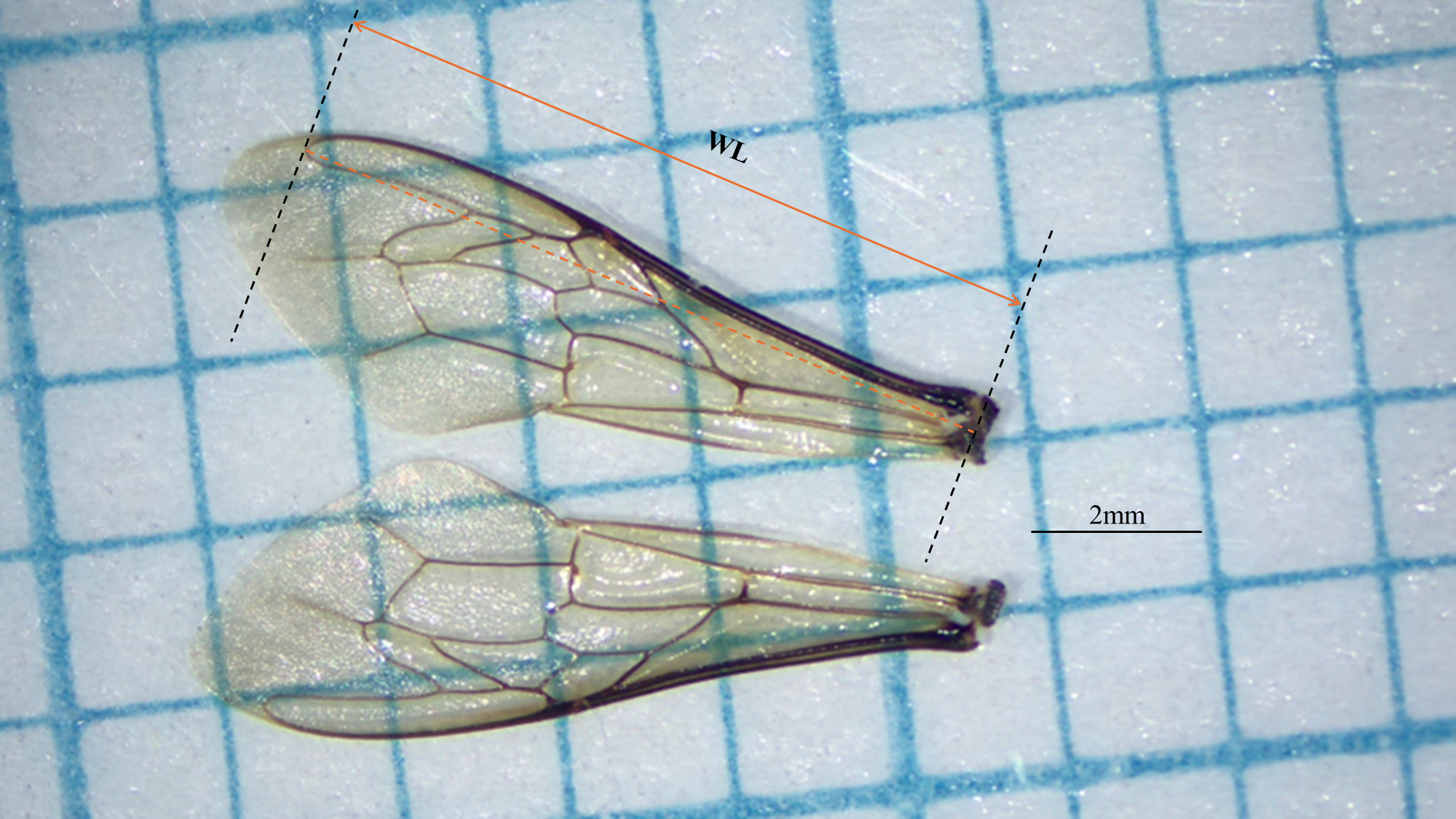


**Fig S3**. The wing length(mm) of the bees (3-03A-01).


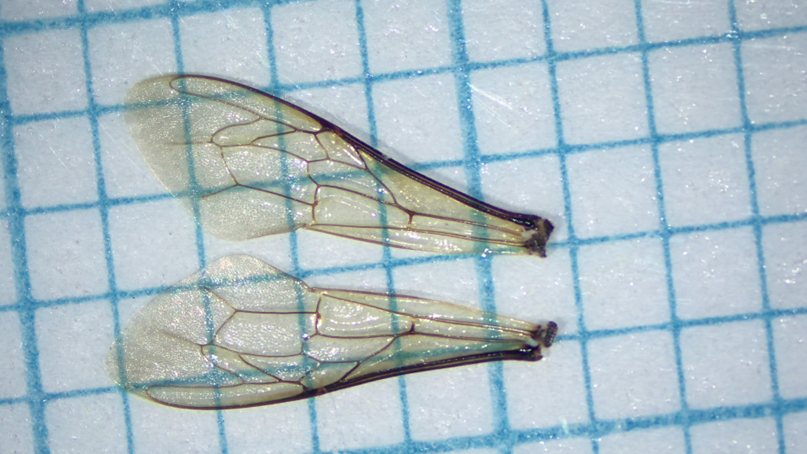


**Fig S4a**. A level: wing margins wear < 10% (3-03A-01).


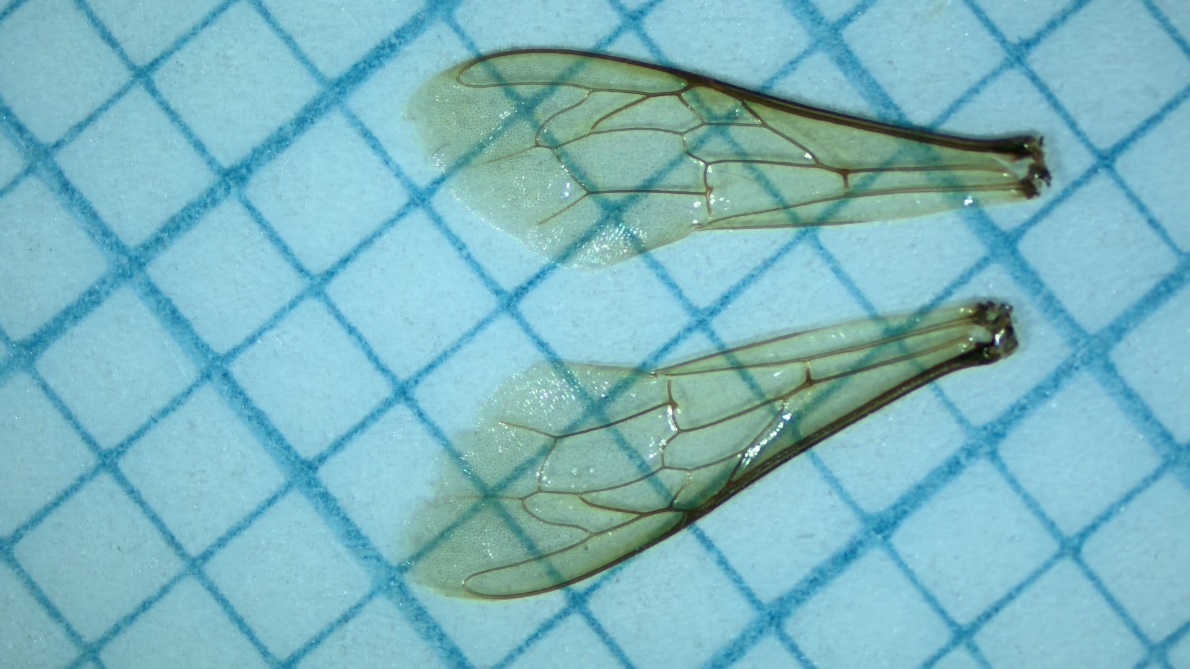


**Fig S4b**. B level: 10% < wing margins wear % < 80% (1-12M-06).


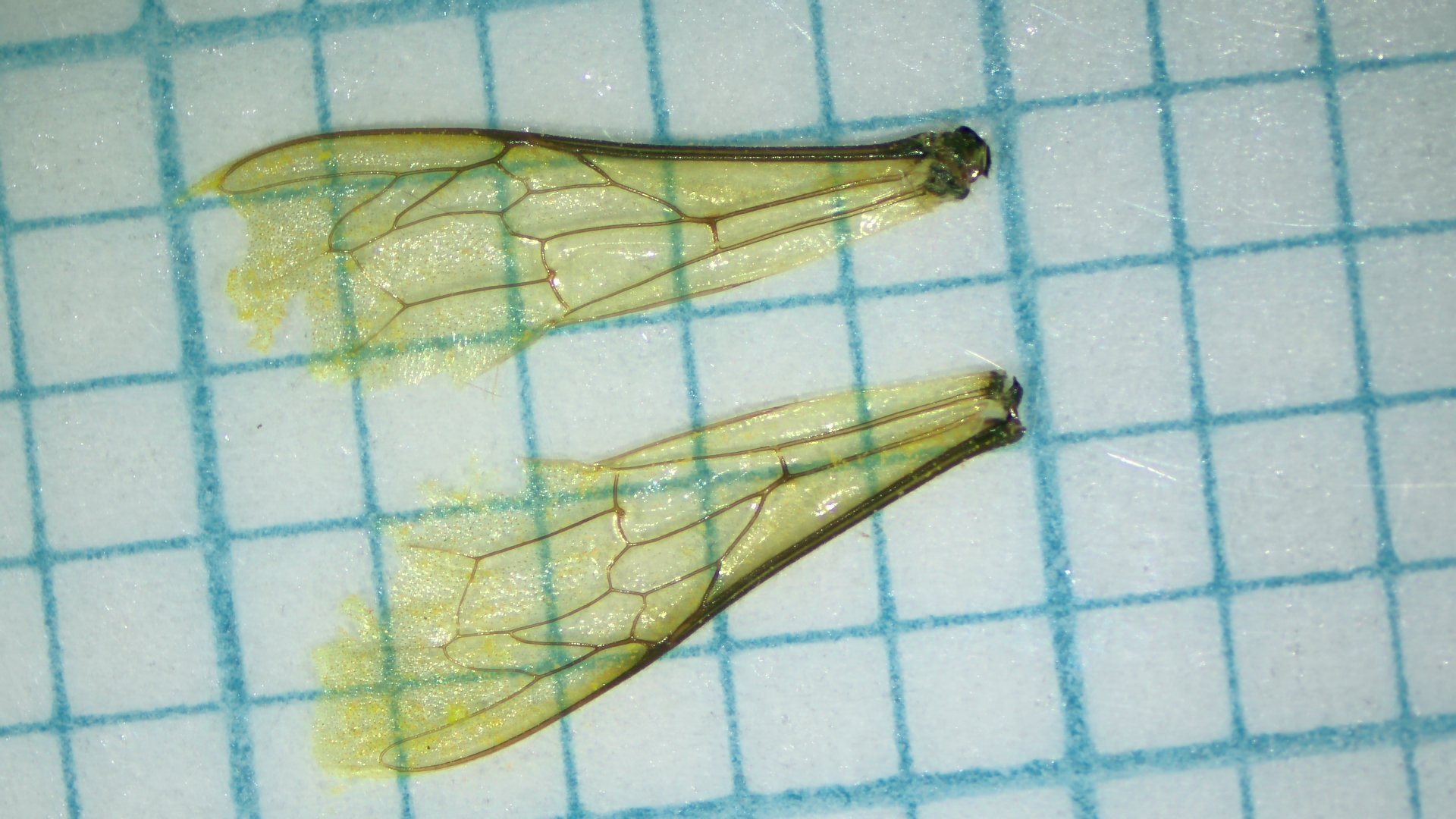


**Fig S4c**. C level: 80% < wing margins wear (1-05M-10).


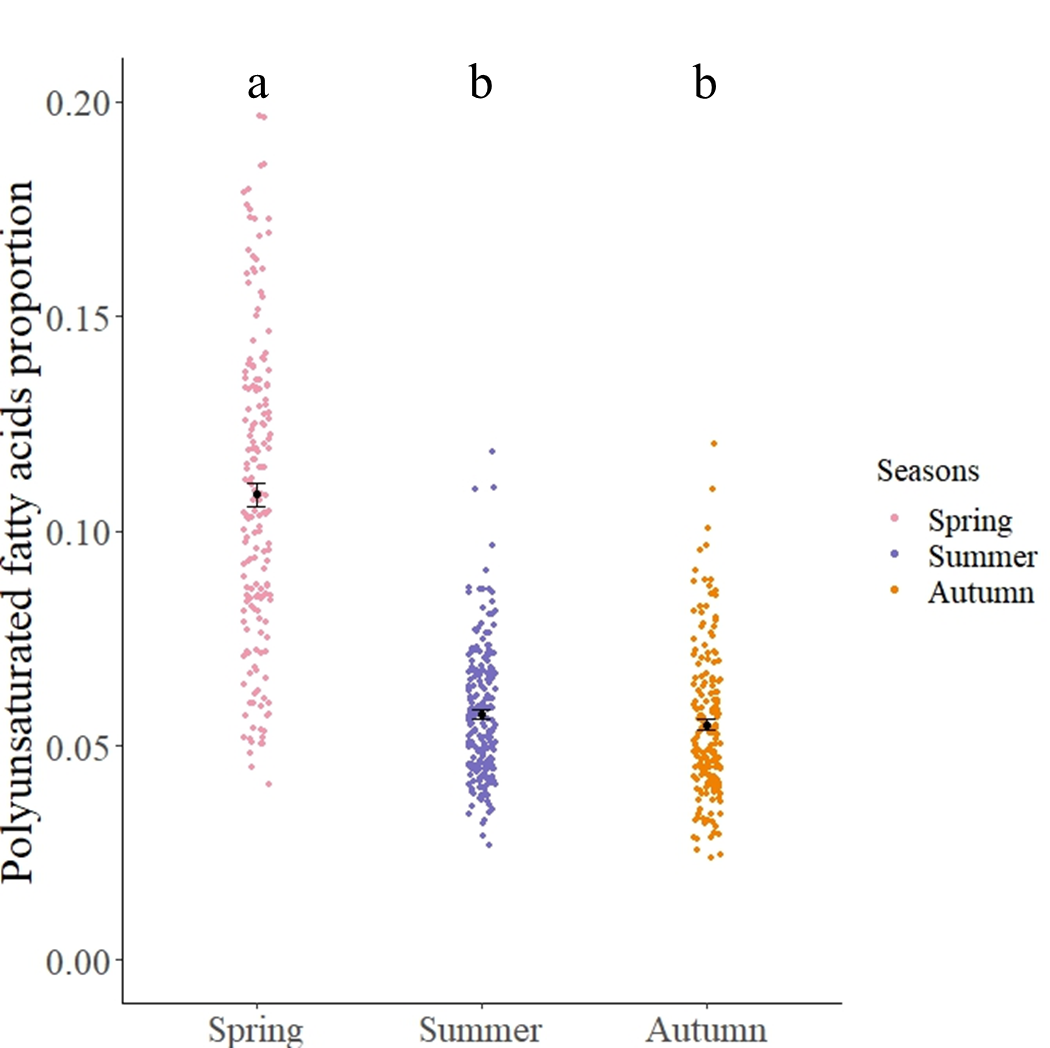


**Fig S5**. Proportion of polyunsaturated fatty acids in captured bee abdomen. Dot and whisker represent the mean, and the standard error. Lowercase letters indicate statistical significance following pair-wise t-test comparisons (p < 0.05).
