# Supplementary material for: Land Use and Season Interactively Affect Honeybee (Apis mellifera) Body Size and Fat Stores
Source: Ecol Evol. 2025 Jul 30;15(8):e71889. doi: 10.1002/ece3.71889 (PMC12310833; doi:10.1002/ece3.71889)
Supplement: Supplementary file 2 — Table S2 Head width (mm) differences in different seasons and landscapes. Table S3. Wing length (mm) differences in different seasons. Table S4. Ratio between wing length and head width in different landscapes and seasons. Table S5. Wing wear differences in different seasons. Table S6. Absolute fatty acid content (AbsFA) in different landscapes and seasons. Table S7. Proportion of each fatty acid content in different seasons. [file ECE3-15-e71889-s001.docx]

**Land use and season interactively affect honeybee (Apis mellifera) body size and fat stores**

| **Table S2. Head width (mm) differences in different seasons and landscapes** | | | | | | | |
| --- | --- | --- | --- | --- | --- | --- | --- |
| Landscapes | Seasons(A) | Season(B) | Difference  of Means | SE | df | t | p |
| Agri | Spring | Summer | 0 | 0.00959 | 267 | -0.586 | 0.8276 |
|  | Summer | Autumn | -0.01 | 0.00954 | 266 | -0.542 | 0.8508 |
|  | Autumn | Spring | 0.01 | 0.00961 | 268 | 1.122 | 0.5014 |
| Urban | Spring | Summer | 0.02 | 0.00972 | 274 | 2.785 | 0.0157 |
|  | Summer | Autumn | -0.03 | 0.00939 | 270 | -3.375 | 0.0024 |
|  | Autumn | Spring | 0.01 | 0.00962 | 269 | 0.479 | 0.8812 |
| Mixed | Spring | Summer | 0.03 | 0.01 | 260 | 3.514 | 0.0015 |
|  | Summer | Autumn | -0.02 | 0.0101 | 261 | -2.007 | 0.1125 |
|  | Autumn | Spring | -0.01 | 0.0101 | 260 | -1.478 | 0.0967 |
| Agri = Agriculture, Urban = Urban, Mixed = mixed habitat | | | | | | | |

| **Table S3. Wing length (mm) differences in different seasons** | | | | | | |  |
| --- | --- | --- | --- | --- | --- | --- | --- |
| Season(A) | Season(B) | Difference of Means | SE | df | t | p |  |
|  |  |  |  |  |  |  |  |
| Spring | Summer | 0.02 | 0.01 | 769 | 1.57 | 0.26 |  |
| Summer | Autumn | 0.01 | 0.01 | 775 | 1.33 | 0.38 |  |
| Autumn | Spring | 0.03 | 0.01 | 773 | -2.85 | 0.01 |  |
|  | | | | | | |  |

| **Table S4. Ratio between wing length and head width in different landscapes and seasons** | | | | | | | |  |
| --- | --- | --- | --- | --- | --- | --- | --- | --- |
| Landscapes | Seasons(A) | Season(B) | Differene  of Means | SE | df | t | p |  |
|  |  |  |  |  |  |  |  |  |
| Agri | Spring | Summer | 0.00986 | 0.00648 | 248 | 1.521 | 0.283 |  |
|  | Summer | Autumn | 0.00922 | 0.00661 | 250 | -1.395 | 0.3451 |  |
|  | Autumn | Spring | -0.01908 | 0.00667 | 251 | -2.862 | 0.0127 |  |
| Urban | Spring | Summer | -0.01625 | 0.00612 | 250 | -2.653 | 0.023 |  |
|  | Summer | Autumn | 0.02258 | 0.00593 | 248 | -3.808 | 0.0005 |  |
|  | Autumn | Spring | -0.00634 | 0.00587 | 247 | -1.08 | 0.5272 |  |
| Mixed | Spring | Summer | -0.00689 | 0.00653 | 233 | -1.055 | 0.5428 |  |
|  | Summer | Autumn | 0.00897 | 0.00669 | 234 | -1.341 | 0.3738 |  |
|  | Autumn | Spring | -0.00208 | 0.00681 | 236 | -0.306 | 0.9497 |  |
| Agri = Agriculture, Urban = Urban, Mixed = mixed habitat | | | | | | | |  |

| **Table S5. Wing wear differences in different seasons** | | | | | |  |
| --- | --- | --- | --- | --- | --- | --- |
| Season(A) | Season(B) | Difference of Means | SE | z | p |  |
|  |  |  |  |  |  |  |
| Spring | Summer | 0.02 | 0.23 | -3.4 | 0.002 |  |
| Summer | Autumn | 0.01 | 0.16 | 4.85 | < 0.001 |  |
| Autumn | Spring | 0.03 | -0 | -0.03 | 1 |  |

| **Table S6. Absolute fatty acid content (AbsFA) in different landscapes and seasons** | | | | | | | | |
| --- | --- | --- | --- | --- | --- | --- | --- | --- |
| Fixed effects | Season(A) | Season(B) | | Differene | SE | df | t | p |
|  |  |  |  | of Means |  |  |  |  |
| Agri | Spring | | Summer | 67 | 46.4 | 172 | 1.451 | 0.3172 |
|  | Summer | | Autumn | -167 | 42.5 | 169 | -3.925 | 0.0004 |
|  | Autumn | | Spring | 100 | 46.9 | 174 | 2.121 | 0.0886 |
| Urban | Spring | | Summer | -67 | 48.4 | 125 | -1.379 | 0.3549 |
|  | Summer | | Autumn | 109 | 38.3 | 170 | 2.8 | 0.0157 |
|  | Autumn | | Spring | -42 | 50 | 134 | -0.834 | 0.6823 |
| Mixed | Spring | | Summer | -104 | 50.9 | 156 | -2.032 | 0.1079 |
|  | Summer | | Autumn | 120 | 46.9 | 165 | 2.553 | 0.0309 |
|  | Autumn | | Spring | -16 | 54.3 | 156 | -0.3 | 0.9517 |
| Spring | Agri | | Mixed | -38 | 66.1 | 48 | -0.579 | 0.832 |
|  | Urban | | Agri | 44 | 67 | 48.4 | 0.656 | 0.7899 |
|  | Mixed | | Urban | -6 | 66.3 | 50.1 | -0.085 | 0.996 |
| Summer | Agri | | Mixed | -217 | 62.5 | 51.4 | -3.467 | 0.003 |
|  | Urban | | Agri | 187 | 62 | 50.4 | 3.011 | 0.0111 |
|  | Mixed | | Urban | 30 | 61.7 | 50.5 | 0.487 | 0.8776 |
| Autumn | Agri | | Mixed | 72 | 51.6 | 45 | 1.164 | 0.4808 |
|  | Urban | | Agri | -90 | 50.3 | 44.1 | -1.492 | 0.3045 |
|  | Mixed | | Urban | 18 | 52 | 44.5 | 0.288 | 0.9554 |
| Agri = Agriculture, Urban = Urban, Mixed = mixed habitat | | | | | | | | |

| **Table S7. Proportion of each fatty acid content in different seasons** | | | | | | | |
| --- | --- | --- | --- | --- | --- | --- | --- |
| Types | Seasons (A) | Seasons (B) | Differene | SE | df | t | p |
|  |  |  | of Means |  |  |  |  |
| SFA | Spring | Summer | 0.003 | 0.00236 | 468 | 1.404 | 0.3394 |
|  | Summer | Autumn | -0.002 | 0.00212 | 510 | -1.065 | 0.536 |
|  | Autumn | Spring | -0.001 | 0.00245 | 479 | -0.433 | 0.9019 |
| PUFA | Spring | Summer | 0.0505 | 0.00256 | 467 | 19.713 | <0.0001 |
|  | Summer | Autumn | 0.0025 | 0.00231 | 510 | 1.086 | 0.5234 |
|  | Autumn | Spring | -0.053 | 0.00266 | 478 | -19.943 | <0.0001 |
| MUFA | Spring | Summer | -0.054 | 0.00392 | 466 | -13.753 | <0.0001 |
|  | Summer | Autumn | -0.00027 | 0.00353 | 511 | -0.076 | 0.9968 |
|  | Autumn | Spring | 0.054 | 0.00407 | 477 | 13.322 | <0.0001 |
| SFA = saturated fatty acids, PUFA = polyunsaturated fatty acids, | | | | | | | |
| MUFA = monounsaturated fatty acids | | | | | | | |
